# Selenium-Dependent Regulation of Oxidative Stress and Immunity in Periparturient Dairy Cattle

**DOI:** 10.1155/2013/154045

**Published:** 2013-01-14

**Authors:** Lorraine M. Sordillo

**Affiliations:** College of Veterinary Medicine, Michigan State University, 784 Wilson Road, East Lansing, MI 48824, USA

## Abstract

Uncontrolled or impaired immune and inflammatory responses in periparturient dairy cows are associated with increased incidence and severity of infectious diseases. The progressive development of oxidative stress during the transition from late gestation to peak lactation is thought to be a significant underlying factor leading to dysfunctional immune cell responses. Certain trace minerals, such as selenium (Se), can ameliorate oxidative stress and reduce the severity of several economically important diseases in dairy cattle including mastitis and metritis. Many of the health benefits of Se can be attributed to the antioxidant functions of selenoproteins. Changes in selenoprotein activity as a consequence of Se nutritional status can directly alter a number of critical cellular functions involved in the inflammatory response. A better understanding of how Se can optimize immune cell responses may facilitate the design of nutritional regimes that will reduce health disorders during the periparturient period.

## 1. Introduction

Dairy cattle have an increased susceptibility to infectious diseases during the periparturient period [1]. A major contributing factor to increased health disorders is thought to be due to dysfunctional bovine immune responses [2, 3]. Indeed, uncontrolled or impaired inflammatory responses are a major contributing factor to several economically important diseases including metritis, laminitis, and mastitis [4]. Increased health problems around the time of calving are especially problematic because they may greatly impact the productive efficiency of dairy cattle in the ensuing lactation. Therefore, it is not surprising that considerable research efforts have focused on defining factors that may contribute to immune dysfunction during this critical period in the production cycle of dairy cows [4–6]. The progressive development of oxidative stress in transition dairy cattle is thought to be a significant underlying factor leading to dysfunctional inflammatory responses [6, 7]. Certain trace minerals, such as Se, can be effective in reducing oxidative stress and the severity of several proinflammatory-based dairy cattle diseases such as mastitis and metritis [6, 8]. Many of the antioxidant functions of Se are mediated through the reducing capacity of selenoproteins including the glutathione peroxidase (GPX) and thioredoxin reductase (TrxR) families. Selenoproteins also can regulate intercellular signaling pathways that orchestrate the expression of mediators that serve to optimize the inflammatory response and restore immune homeostasis. A better understanding of how Se can optimize bovine immune responses during the transition period may facilitate the design of nutritional regimes that will reduce the severity and duration of disease as a function of dysfunctional inflammatory responses. This paper will describe the role of reactive oxygen species in regulating immune cell populations and how oxidative stress during the periparturient period can adversely affect dairy cattle immunity. The benefits that adequate Se nutritional status can have in controlling oxidative stress and improving immune responses of dairy cattle during the periparturient period will be discussed.

## 2. Impact of Reactive Oxygen Species on Immunity

Innate and acquired immune defenses of dairy cattle are compromised during the periparturient period, and several recent reviews summarize these changes in considerable detail [5, 9–11]. Dairy cattle undergo dramatic metabolic changes during the periparturient periods that likely contribute to dysfunctional immune responses and increased disease susceptibility [4, 6]. Following calving, there is a dramatic increase in energy utilization needed to support the onset of copious milk synthesis and secretion. The conversion of nutrients into an energy source needed to fuel normal physiological functions occurs through a set of metabolic reactions collectively referred to as cellular respiration. Oxygen is required for aerobic cellular respiration, and reactive oxygen species (ROS) are metabolites formed in the mitochondria during this process as byproducts of the electron transport chain reaction [12]. Although the majority of ROS found in most tissues results from increased cellular metabolism and energy generation, other potential sources include various oxidizing enzyme pathways including those associated with host immune responses [13].

### 2.1. Immunological Role of ROS

ROS are potent metabolites that can increase the oxygenation of other molecules involved in regulating important cellular functions such as differentiation and proliferation [12]. The production of low or moderate concentrations of ROS is especially essential for a number of normal processes related to innate and acquired immune responses. Phagocytosis is an essential component of the cellular innate immune response, for example, that involves the generation of toxic ROS necessary for the oxygen-dependent destruction of invading pathogens. Macrophages and neutrophils first engulf microbial pathogens, followed by the formation of an intracellular phagosome. The NADPH oxidase system is localized within the phagosomal membrane and once activated, is responsible for generating the majority of ROS that can destroy the engulfed pathogen [14]. Superoxide anion (O_2_^•−^) radicals are generated initially by NADPH oxidase, but these ROS have very little bactericidal activity. In the presence of superoxide dismutase, however, O_2_^•−^ is converted into a more potent hydrogen peroxide (H_2_O_2_). Myeloperoxidase can then catalyze a reaction between H_2_O_2_ and chloride ions to generate the highly toxic hypochlorous acid (HOCl) which is considered the major oxidative compound used to kill engulfed pathogens [14]. 

Ample evidence also suggests that some ROS are involved in signal transduction pathways leading to the expression of cytokines, eicosanoids, and other immunoregulatory factors essential to host defense during infection [15–19]. Certain ROS, such as H_2_O_2_, can diffuse out of the mitochondria and into the cytoplasm where it can interact with several targets involved in cell signaling pathways. Nuclear factor-(NF-)*κ*B is a dimeric transcription factor that regulates a large number of genes involved in controlling many aspects of immune and inflammatory responses. Normally localized in the cytoplasm, NF-*κ*B will become activated and translocate to the nucleus in response to ROS accumulation [20]. Activated NF-*κ*B can then interact with gene promoters in the nucleus resulting in the increased expression of cytokines, chemokines, and other factors involved in the inflammatory response [16]. The mitogen-activated protein kinases (MAPKs) pathway is also responsive to ROS activation and involved in regulating genes associated with the immune and inflammatory response. Both p38 and JNK become phosphorylated when the kinase apoptosis-signal kinase-1 (ASK-1) is activated through ROS-mediated oxidation [21]. Therefore, intracellular ROS are critical for activating several key redox-regulated signaling pathways that orchestrate host immune responses to invading pathogens. There is even evidence to suggest that the ROS generated by NADPH oxidase during phagocytosis may contribute to host defense not only by direct bactericidal actions, but also by modulating some of these ROS-sensitive pathways in phagocytic leukocytes [22].

Finally, ROS can control the magnitude and duration of the inflammatory response by altering the function of vascular endothelial cells [18, 19]. The vascular endothelium has an important role in the inflammatory process due to its strategic location between the blood and underlying infected or damaged tissue. The initiation and resolution of the inflammatory response depend to a great extent on vascular tone and expression of adhesion molecules on endothelial cells that line the blood vessel wall. Changes in vascular tone are essential for increasing blood flow and blood-derived immune components to localized areas of infection. There is considerable evidence to show that changes in the amount of endothelial cell-derived ROS play a central role in regulating vasoconstriction and vasodilation during an inflammatory response [18, 19, 23]. Increased expression of vascular adhesion molecules is important for directing peripheral blood leukocytes to the underlying infected tissues. The recruitment of neutrophils and monocyte is dependent, in part, on the progressive activation of the endothelium and the sequential expression of several different adhesion molecules. The selectin family of adhesion molecules, including E-selectin and P-selectin, are primarily responsible for the initial phase of the leukocyte adhesion cascade. The Ig superfamily of adhesion molecules includes intercellular adhesion molecule-1 (ICAM1), vascular adhesion molecule-1 (VCAM1), and platelet endothelial adhesion molecule-1 (PECAM1) [24]. The expression of both ICAM1 and VCAM1 increase significantly during inflammation and is involved in the firm attachment of blood leukocytes to vascular endothelial cells [18]. The PECAM1 is primarily associated with endothelial cell intercellular junctions and is important in regulating transmigration of leukocyte across the endothelium [18, 24]. Sufficient evidence exists to indicate the important role that ROS play in regulating the synthesis and surface density of adhesion molecules on endothelial cells, and therefore, ROS are essential for optimizing inflammatory responses especially during the early stages of disease [18, 19, 25].

### 2.2. Oxidative Stress

Whereas ROS have numerous beneficial effects on immune and inflammatory responses, damage to host cells can occur if buildup of these highly reactive molecules becomes excessive. Although small fluctuations in the steady-state concentrations of ROS are necessary for optimal immune and inflammatory responses, dramatic imbalances can result in tissue damage and loss of normal cell function [6, 13, 26, 27]. Oxidative stress is a term used to describe various deleterious processes resulting from an imbalance between excessive formation of ROS and/or reduced antioxidant defenses [28]. Several endogenous antioxidant defense mechanisms are present to tightly regulate ROS accumulation within tissues [13]. Antioxidant defenses are capable of slowing or preventing the oxidation of other molecules and can be characterized as either radical scavengers or detoxifying enzyme systems [29]. Disturbances in the balance between ROS production and antioxidant defenses can result in substantial damage to nearby tissues by oxidizing cellular lipids, proteins, and DNA. Membrane phospholipids, for example, are especially susceptible to peroxidation and the subsequent formation of lipid radicals. If allowed to accumulate, these lipid peroxy radicals can act on adjacent fatty acids in the cellular plasma membranes and induce even more radical formation through positive feedback loops. As a result, excess ROS accumulate and can lead to a loss of normal membrane function and even cell death if the condition persists [28, 29]. 

In the periparturient cow, tissues consume more oxygen through normal cellular respiration during times of increased metabolic demand in order to provide the energy needed for the onset of lactation. This increase in metabolic activity results in the enhanced accumulation of ROS and the depletion of important antioxidant defenses around the time of calving [6, 7, 30–32]. The natural balance between ROS formation and antioxidant defense can be disrupted further by several other factors including disease challenge, obesity, increased plasma nonesterified fatty acid concentrations, and environmental stress (i.e., heat stress) [30, 33–36]. Moreover, the cow's antioxidant response to oxidative stress requires energy that could be better used for production. As a result, increased oxidative stress due to excessive accumulation of ROS can diminish the productive efficiency of periparturient cows. Antioxidant defenses are diverse, can be either synthesized in the body or derived from the diet, and are localized transiently throughout tissues and different cell types. Trace minerals are an important source of dietary-derived antioxidants and are known to play an important role in optimizing bovine immune responses and disease resistance [8, 37]. The health benefits of Se, for example, are thought to be mediated by Se-containing antioxidant enzymes that prevent oxidative stress by reducing ROS to less reactive molecules, thus restoring an appropriate balance of reduced and oxidized molecules within cells [11]. The importance of Se in the diet of dairy cattle is especially well-documented based on its ability to reduce the incidence and severity of disease during times of heightened oxidative stress [6, 8]. 

## 3. Se and Dairy Cattle Health

Se is an essential trace mineral in dairy cattle that is required to maintain normal physiological functions and provides a significant dietary source of antioxidant defenses. The importance of Se on the health and productivity of dairy cattle is well documented in the literature [38]. The most severe cases of Se deficiency in ruminants can result in nutritional myopathies referred to as white muscle disease [39]. Marginal Se deficiency is more commonly observed in the adult dairy cattle population and is considered an important risk factor for mastitis, retained fetal membranes, and metritis in the periparturient period [8, 38, 40, 41]. The significance of Se in the health of dairy cows is best illustrated in the severity and duration of mastitis. Over two decades ago, researchers showed that Se deficiency in cows was associated with higher milk somatic cell counts (SCCs) and lower resistance to clinical mastitis during early lactation [42, 43]. In addition, higher concentrations of Se in the plasma of cows were negatively correlated to bulk tank SCC [44]. More recent studies confirmed that higher bulk tank Se concentrations were associated with a lower risk of being a *Staphylococcus aureus*-positive herd [45]. Moreover, Se supplementation of pastured dairy heifers and cows before calving reduced the prevalence of new intramammary infections and high SCC during early lactation [46, 47]. Se nutritional status is important to many reproductive functions of dairy cattle as well. Research has shown that Se supplementation of otherwise Se-deficient dairy cows can reduce the number of services per conception, improve pregnancy rates at first service, and result in fewer days to conception [41]. Se supplementation also was effective in reducing the incidence of metritis and cystic ovaries during the early postpartum period [48, 49]. The major beneficial health effects of Se are thought to be a function of supporting important antioxidant enzyme systems and controlling oxidative stress. Indeed, several studies have shown that adequate Se supplementation can reduce oxidative stress especially in high producing dairy cattle during the periparturient period [8, 50, 51]. However, recent information related to both human and veterinary medicine suggests that the role of Se in controlling health disorders may be more complex than only through its antioxidant functions [52–54].

## 4. Se and Immune Cell Functions

The beneficial health effects derived from adequate Se nutrition have been attributed to the impact of this trace mineral on dairy cattle immune cell functions [6, 55]. The innate immune response plays an important role in preventing the establishment of infections. Indeed, the ability of neutrophils to rapidly migrate into mammary tissues and to effectively kill invading pathogens is a major factor that determines the establishment of new intramammary infections [56]. Early studies showed that Se deficiency in dairy cows reduces the ability of both blood and milk neutrophils to kill mastitis-causing pathogens [57, 58]. The addition of Se to neutrophils *in vitro*, however, was effective at enhancing the chemotactic migration and increased the production of superoxide needed for bactericidal activity [59]. Neutrophils obtained from cows with higher blood concentrations of Se also had a greater potential to produce superoxide and kill bacterial pathogens [60]. Macrophages are a dominant leukocyte type found in healthy mammary glands and represent another important early defense mechanism during the early stages of infection. During mastitis, macrophages function by not only phagocytizing bacteria, but also by releasing cytokines and eicosanoids that facilitate the migration and bactericidal activities of neutrophils [5, 56]. Studies showed that *in vitro* supplementation of mammary gland macrophages with Se enhanced the production of neutrophil chemotactic factors following stimulation with *S. aureus* [61]. Lymphocytes also can play an important role in regulating cellular immunity through the production of immunoregulatory factors following stimulation. Peripheral blood lymphocytes isolated from Se-deficient dairy cattle had reduced rates of mitogen-induced proliferation and reduced eicosanoid biosynthesis by way of the 5-lipoxygenase (LOX) pathway when compared to Se-sufficient cows [62]. Improvements in lymphocyte proliferative responses were reported when lymphocyte cultures were supplemented *in vitro* with increasing dose of Se [61]. Cao et al. suggested that the reduced production of 5-LOX metabolites may be a causative factor in decreased lymphocyte proliferation and may contribute to decreased disease resistance in Se-deficient animals [62]. 

The vascular endothelium has received relatively little research attention concerning bovine mastitis and other inflammatory-based diseases of dairy cattle even though endothelial cells play a critical role in host inflammatory responses. Endothelial cells orchestrate inflammatory responses in various ways including changes in vascular tone and blood flow to accommodate leukocyte slowing and migration from the blood and into the underlying infected tissue. Endothelial cells also express adhesion molecules and are a source and cellular target for pro-inflammatory cytokines and vasoactive eicosanoids [5]. Se nutritional status can directly influence vascular endothelial cell functions in several ways. Bovine mammary and aortic endothelial cells grown in Se-deficient culture media exhibited increased platelet activating factor (PAF) biosynthesis [63, 64], and increased PAF expression is associated with increased vascular disorders during oxidative stress [65]. Se status also can modify the biosynthesis of other vasoactive lipid mediators such as the eicosanoids. Se-deficient bovine endothelial cells altered the profile of arachidonic acid metabolism by both the COX and LOX pathways [66, 67]. Compared with Se adequate endothelial cells, the production of prostaglandin (PG) I_2_, PGF_2*α*_, and PGE_2_ was significantly decreased in the Se-deficient endothelial cells [66]. Se deficiency, however, significantly increased the biosynthesis of thromboxane B_2_ (TXB_2_) and 15-hydroperoxyeicosatetraenoic acid (15-HPETE) that is associated with the pathophysiology of several inflammatory-based diseases in human [66, 67]. Indeed, the enhanced production of 15-HPETE was shown to be the major causative factor contributing to endothelial cell apoptosis during Se deficiency [68]. Similar changes in milk eicosanoid concentration also were reported in Se-deficient dairy cows with coliform mastitis suggesting that shifts in eicosanoid profiles may be associated with the altered pathogenesis and outcome of mastitis during Se deficiency [25]. 

Delayed neutrophil migration is associated with the severity of coliform mastitis [69] and Se supplementation increased the speed of neutrophil migration into bovine mammary glands during an *Escherichia coli* infection [70]. The overexpression of certain vascular adhesion molecules, such as ICAM1, is associated with the pathophysiology of inflammatory-based diseases in humans possibly due to the disruption in leukocyte transmigration responses [71]. Bovine mammary endothelial cells cultured in Se-deficient media exhibited enhanced ICAM1 expression and increased neutrophil adherence when stimulated with tumor necrosis factor or H_2_O_2_ suggesting one mechanism for the delayed migration of leukocytes into infected tissues [24, 72]. Although Se status is closely linked with the function of cells involved in immune and inflammatory responses, the precise mechanisms responsible for the beneficial effect of Se are not fully understood.

### 4.1. Functions of Se and Selenoproteins

Many of the positive biological effects of Se are thought to be due to its incorporation into a family of proteins called selenoproteins. Se is incorporated into selenoproteins as a selenocysteine (Sec) residue [73, 74]. A unique mechanism of Sec incorporation exists where a UGA codon in the mRNA of selenoproteins is utilized to cotranslationally incorporate Sec into the growing polypeptide. To date, there are 25 different selenoproteins genes in humans that have been identified and characterized to a limited extent. Selenoproteins can optimize immune and inflammatory responses in several different ways such as reducing toxic ROS to less reactive molecules, modifying the enzymes involved in eicosanoid biosynthesis, and regulating intercellular signaling pathways that lead to inflammatory gene expression [52].

Most antioxidant functions of Se can be attributed to the glutathione peroxidases (GPXs) and thioredoxin reductases (TRxRs), where Sec residues are located in the active site required for catalytic activity. In dairy cattle, however, the most widely studied selenoprotein is cytosolic GPX1. Previous studies suggest that the antioxidant functions of GPX1 are the primary reason why Se improves bovine innate immune responses [8, 50]. Se, through the actions of GPX1, is thought to protect phagocytic cells from oxidative damage that may occur during respiratory burst. Leakage of ROS from the phagosome or failure to reduce ROS to less reactive metabolites could cause bystander damage to neutrophils and result in a reduction of bactericidal functions [75]. Several studies have shown a negative correlation that exists between whole blood GPX1 activity and bulk tank milk SCC [44, 76]. Higher blood GPX activity following Se supplementation also was correlated to reduced prevalence of new intramammary infections in pastured heifers [46]. Less is known concerning the role of other selenoproteins in bovine immune and inflammatory responses. Recent evidence suggests that decreases in TRxR activity of bovine peripheral blood mononuclear cells are associated with increased oxidative stress in periparturient dairy cows [36]. Strong positive correlations were observed between gene expression of GPX1, GPX4, and TRxR with vascular adhesion molecules in mammary tissue samples obtained during the periparturient period suggesting a potential protective response from all these antioxidant enzymes during oxidative stress [77]. Increased selenoprotein activity following *in vitro* supplementation of bovine endothelial cells with Se also was associated with less oxidant-induced inflammation and apoptosis [68, 72]. Silencing specific selenoproteins activity with siRNA suggested that TRxR is especially important in protecting bovine endothelial cells from the deleterious effects of prooxidant challenge [78, 79].

Selenoproteins not only function as antioxidant enzymes, but also in thyroid hormone metabolism, redox signaling, and regulation of immune responses. Iodothyronine deiodinases, for example, are important in the regulation of thyroid hormone expression and metabolic functions [52, 80]. Many studies have been conducted in models of human diseases to illustrate the importance of individual selenoproteins in the redox regulation of inflammatory signaling pathways and in regulating the functions of immune cells other than through control of oxidative stress [52]. Considerably less is known about the cellular and molecular mechanisms involved in the functions of selenoproteins in the bovine immune system. A better understanding of the specific cell signaling pathways and immune responses regulated by dietary Se may lead to more consistent improvements in dairy cattle health and performance in the periparturient period.

## 5. Conclusion

The concentration of Se in the soil can vary greatly depending on geographical location, and it follows that crops grown on these soils would also vary in Se content. In many dairy-intensive regions, however, soils tend to be very low in Se content, and cows must be supplemented with Se to avoid nutritional deficiencies. The National Research Council has set the Se requirement for all dairy cattle at 0.3 ppm [81]. The 0.3 ppm target is based primarily on providing enough Se to prevent measurable deficiencies, but not necessarily to optimize animal health or prevent toxicity due to over consumption. Even with the widely accepted practice of supplementing dairy rations with Se and other trace minerals, oxidative stress and associated health disorders continue to be a problem in periparturient cows. Given the importance of Se in optimizing host immune and inflammatory responses, there is a need to find more efficient ways of improving the Se status of cows without exceeding the legal limits on supplemental Se. One approach is examining the benefits of using organic over inorganic sources of Se to improve absorption and retention in the body. The limited clinical data comparing Se sources, however, suggests that organic forms have little benefit over inorganic sources of Se on the functional capabilities of blood neutrophils or reducing mastitis [55, 82]. Factors affecting Se bioavailability in ruminants are poorly understood, and better methods for assessing Se nutrition status with functional and relevant biomarkers is a major unfilled need for improving health outcomes. Additional information of how different chemical forms of Se are absorbed and retained in targeted tissue of cows is needed to optimize dietary Se supplementation. Moreover, very little is known concerning the role of individual selenoproteins and/or other intermediate Se metabolites in orchestrating host immune and inflammatory responses during time of oxidative stress. A better understanding of the cellular and molecular actions of selenoproteins may lead to novel targeted therapies that can improve the health and well-being of the periparturient dairy cow. 
